# Metagenomic Next-Generation Sequencing Reveals Individual Composition and Dynamics of Anelloviruses during Autologous Stem Cell Transplant Recipient Management

**DOI:** 10.3390/v10110633

**Published:** 2018-11-14

**Authors:** Antonin Bal, Clémentine Sarkozy, Laurence Josset, Valérie Cheynet, Guy Oriol, Jérémie Becker, Gaëlle Vilchez, Pierre Sesques, François Mallet, Alexandre Pachot, Florence Morfin, Bruno Lina, Gilles Salles, Fréderic Reynier, Sophie Trouillet-Assant, Karen Brengel-Pesce

**Affiliations:** 1 Laboratoire de Virologie, Institut des Agents Infectieux, Groupement Hospitalier Nord, Hospices Civils de Lyon, 69004 Lyon, France; laurence.josset@chu-lyon.fr (L.J.); florence.morfin-sherpa@chu-lyon.fr (F.M.); bruno.lina@chu-lyon.fr (B.L.); 2 Univ Lyon, Université Lyon 1, Faculté de Médecine Lyon Est, CIRI, Inserm U1111 CNRS UMR5308, Virpath, 69372 Lyon, France; sophie.assant@chu-lyon.fr; 3 Laboratoire Commun de Recherche HCL-bioMerieux, Centre Hospitalier Lyon Sud, 69495 Pierre-Bénite, France; valerie.cheynet@biomerieux.com (V.C.); guy.oriol@biomerieux.com (G.O.); gaelle.vilchez@ext.biomerieux.com (G.V.); francois.mallet@biomerieux.com (F.M.); alexandre.pachot@biomerieux.com (A.P.); karen.brengel-pesce@biomerieux.com (K.B.-P.); 4 Service d’Hématologie Clinique, Centre Hospitalier Lyon Sud, Pierre-Bénite, 69495 Lyon, France; clementine.sarkozy@chu-lyon.fr (C.S.); pierre.sesques@chu-lyon.fr (P.S.); gilles.salles@chu-lyon.fr (G.S.); 5 Bioaster, Genomics and Transcriptomics Technological Unit, 69007 Lyon, France; jeremie.becker@bioaster.org (J.B.); frederic.reynier@bioaster.org (F.R.)

**Keywords:** next-generation sequencing, viral metagenomics, immunocompromised patients, stem cell transplant recipients, *Anelloviridae*, torque teno virus

## Abstract

Over recent years, there has been increasing interest in the use of the anelloviruses, the major component of the human virome, for the prediction of post-transplant complications such as severe infections. Due to an important diversity, the comprehensive characterization of this viral family over time has been poorly studied. To overcome this challenge, we used a metagenomic next-generation sequencing (mNGS) approach with the aim of determining the individual anellovirus profile of autologous stem cell transplant (ASCT) patients. We conducted a prospective pilot study on a homogeneous patient cohort regarding the chemotherapy regimens that included 10 ASCT recipients. A validated viral mNGS workflow was used on 108 plasma samples collected at 11 time points from diagnosis to 90 days post-transplantation. A complex interindividual variability in terms of abundance and composition was noticed. In particular, a strong sex effect was found and confirmed using quantitative PCR targeting torque teno virus, the most abundant anellovirus. Interestingly, an important turnover in the anellovirus composition was observed during the course of the disease revealing a strong intra-individual variability. Although more studies are needed to better understand anellovirus dynamics, these findings are of prime importance for their future use as biomarkers of immune competence.

## 1. Introduction

Autologous stem cell transplant (ASCT) is the cornerstone of treatment for certain hematological malignancies such as multiple myeloma (MM) or lymphoma. The high-dose chemotherapy administrated prior to the graft lead to an immunosuppression state associated with a high rate of infections responsible for treatment-related morbidity and mortality [1]. Clinical patient management urgently needs an immune system biomarker that could be helpful to predict these complications and help in the transplant decision or to adapt antimicrobial prophylaxis. The blood viral load monitoring of the torque teno virus (TTV), a virus belonging to the *Anelloviridae* family, is increasingly proposed in this setting as illustrated by their use for the risk prediction of graft rejection and bacterial infections in solid organ transplant patients [2,3,4]. TTV is a member of *Alphatorquevirus* genus which includes 29 species grouped in five genogroups [5,6]. Human *Anelloviridae* are also represented by torque teno mini virus (TTMV) and torque teno midi virus (TTMDV) belonging to *Betatorquevirus* and *Gammatorquevirus* genera, respectively [5]. Hence, the current focus on TTV using targeted polymerase chain reaction (PCR) does not reflect the very high genetic diversity of this viral family. As it is an assumption-free technique, viral metagenomics next-generation sequencing (mNGS) is adapted to fully explore anellovirus diversity, but also to the detection of novel species [7]. Furthermore, some authors have suggested that not all anelloviruses species have the same interactions with the host immune system and therefore the same potential as a biomarker [5,8]. While coinfections with multiple anelloviruses are highly frequent in populations with hematological diseases [9], their detailed composition and dynamics during the clinical course of the disease are still poorly described. The objective of this prospective pilot study was to determine the individual plasma anellovirus composition and abundance over time in a cohort of ASCT recipients using mNGS.

## 2. Materials and Methods

### 2.1. Study Population

As chemotherapy may impact differentially virome composition and abundance, 10 multiple myeloma patients (MM) receiving the same chemotherapy regimen were studied. It included four cycles of VTD (velcade, thalidomide, dexamethasone) for induction therapy; high-dose melphalan was used as a conditioning regimen, and, for maintenance therapy two VTD cycles were administered. MM patients were recruited prospectively from November 2015 to October 2017 in the hematology department of the Lyon-Sud University Hospital, Lyon, France. The median age was 58 years (range: 51 to 66) and the sex ratio (M/F) was 4/6. Five patients developed an infection post-ASCT including four bacteremia (three *E. coli* and one *S. pneumoniae*) and one cytomegalovirus reactivation. Metagenomic NGS testing was performed on plasma samples collected at 11 times points from hematological malignancy diagnosis to 90 days post-ASCT. The median duration of follow-up was nine months (range: 8 to 9). Eight plasma samples matched on age and sex from healthy volunteers (HV) were also sequenced.

### 2.2. Metagenomic Workflow and Bioinformatic Analysis

A validated viral metagenomics process including quality controls was used as previously described [10]. Briefly, after sample viral enrichment, total nucleic acid was extracted, randomly amplified, and Illumina libraries were prepared using the Nextera XT DNA Library preparation kit according to the manufacturer’s recommendations (Illumina, San Diego, CA, USA). Twelve samples per run were sequenced on the Illumina NextSeq500™ platform. In each run, a no-template control (NTC) was implemented to evaluate the contamination during the workflow. This NTC (nuclease-free water) was processed through all mNGS steps. A stepwise bioinformatics pipeline was used. Low quality and human reads were filtered out using cutadapt and bwa mem on the human genome (GRCh37.p2), respectively. Remaining reads were mapped on viral RefSeq database (downloaded in March 2015) to determine the relative abundance of anelloviruses within the plasma virome. As recommended, a BLAST-based validation of metagenomic sequence assignments was performed for anelloviruses [11]. Thus, nonhuman NGS reads were aligned using BLAST on a manually curated database composed of the 56 reference sequences of human anelloviruse species as established by the International Committee on Taxonomy of Viruses (ICTV, TTV-1 to TTV-29, TTMV-1 to TTMV-12, TTMDV-1 and TTMDV-15). To clarify the representation of the individual dynamics for TTV, the number of reads obtained for each TTV species were grouped in TTV genogroups (TTV 1 to TTV 5) [6,12]. Of note, a cut-off value of 35% nucleotide sequence identity was determined by ICTV as a specie demarcation criterion (based on the open reading fram 1 analysis). Concerning the TTV genogroups, the sequence divergence was estimated at 45% [12]. To correct differences in sample sequencing depth, the number of reads were normalized using reads per million mapped reads (RPM) ratio. The sequence data generated in this study has been deposited in the Sequence Read Archive (BioProject: PRJNA504035)

### 2.3. TTV Quantification

TTV quantification was performed with a standardized quantitative PCR (qPCR) on all samples tested with mNGS [13]. Briefly, TTV DNA was extracted from 200 μL of plasma using the NucliSENS easy MAG platform (bioMérieux, Marcy l’Etoile, France) and eluted in 50 μL of elution buffer. TTV DNA was quantitated by TaqMan real-time PCR according to the manufacturer’s recommendations (TTV R-gene, bioMérieux), and results were recorded as copies/mL. The claimed limit of detection was 2.2 log_10_ copies/mL in plasma.

### 2.4. Statistical Analysis

Statistical analyses were conducted using GraphPad Prism^®^ software (version 5.02; GraphPad software, La Jolla, CA, USA). Correlations between mNGS and qPCR assays were determined by applying the Pearson’s correlation coefficient. All comparisons between median values were performed using the nonparametric Mann–Whitney test. Differences were considered significant at *p* < 0.05.

### 2.5. Ethics Statement

This non-interventional study received authorization from the French data protection body (Commission Nationale de l’Informatique et des Libertés—CNIL—agreement n° DR-2015-694) and was approved by the ethics committee (Comité consultatif sur le traitement de l’information en matière de recherche—CCTIRS, Paris, France—agreement n°15-529). All patients gave written informed consent.

## 3. Results

### 3.1. Anellovirus Dynamics within the Plasma Virome

**Figure 1:**
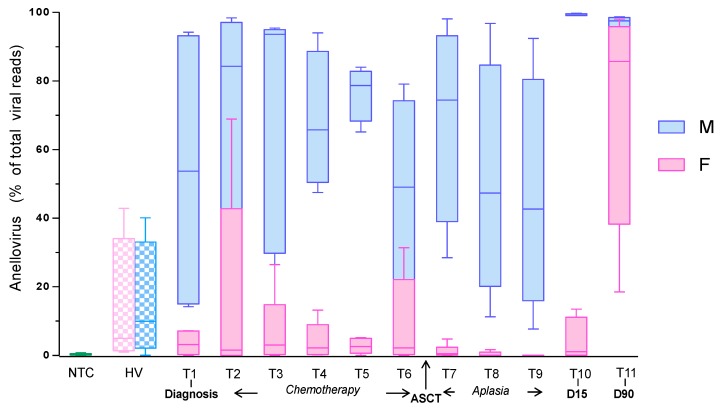
Proportion of anellovirus reads in NTC, HV, and ASCT recipients. The median of anellovirus reads proportion (among total viral reads) obtained from the 10 ASCT recipients and 8 HV is represented according to gender. One NTC (nuclease free-water processed through the metagenomics workflow) per batch was used to evaluate the contamination. The median value of anellovirus is also represented for the NTC. NTC: no-template control; HV: healthy volunteers; ASCT: autologous stem cell transplantation; F: female; M: male.

Longitudinal plasma samples (*n* = 108) from 10 MM patients were collected and tested using mNGS. A median of 28,210,575 reads per sample was generated, of which a median of 0.1% were of viral origin. The anellovirus contamination represented only 0.2% of the NTC viral reads. Strikingly, the overall relative abundance of anellovirus was significantly higher in men than women for MM patients (78.3% vs. 1.2% of the total viral reads, respectively, *p* < 0.001); a trend towards this was found in HV (10.0% vs. 5.0%, *p* = 0.89; Figure 1). In MM patients, both among men and women the lowest level was found during aplasia (44.7% and 0.1%, respectively) and a peak occurred at 15 days post-ASCT (99.2%) among men, and at 90 days among women (85.7%; Figure 1; Figure S1).

### 3.2. Anellovirus Abundance and Composition among Individuals

**Figure 2:**
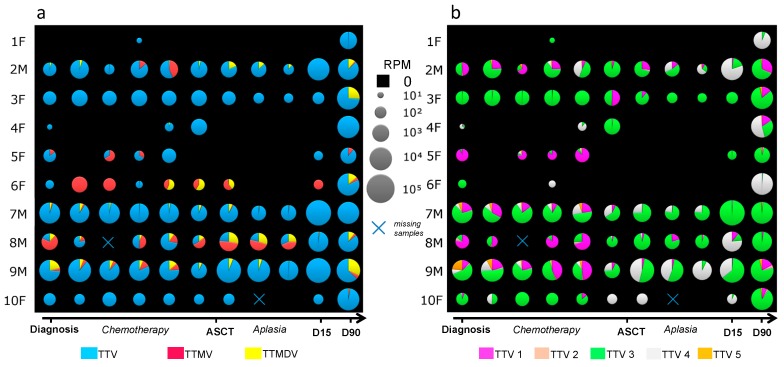
Anellovirus abundance and composition among the 10 ASCT recipients from diagnosis to 90 days post-ASCT (**a**) Kinetics of TTV, TTMV, and TTMDV (**b**) Kinetics of the 5 TTV genogroups. TTV: torque teno virus; TTMV: torque teno mini virus; TTMDV: torque teno midi virus; ASCT: autologous stem cell transplantation; F: female; M: male. RPM: reads per million mapped reads.

Anellovirus reads were detected in 84/108 (77.8%) MM samples. TTV was the most abundant (91.7% of anellovirus reads), while TTMDV and TTMV represented 6.4% and 1.9%, respectively. Mixed infections with at least 2 genera were found in 52/84 (61.9%) anellovirus-positive samples. Of note, TTMDV was the main genus detected in HV samples (49.5% of HV anellovirus reads). Anellovirus reads were detected at each time point including the aplasia period for 5/10 patients (Figure 2a). Concerning the distribution of TTV genogroups, TTV 3 was predominant (67.1% of TTV reads) followed by TTV 4 and TTV 1 (23.9 % and 6.5%, respectively; Figure 2b). Among them, the species TTV-16, TTV-15 (TTV genogroup 3) and TTV-29 (TTV genogroup 4) represented more than 50% of all anellovirus reads detected in the 108 MM samples (28.8%, 8.2% and 20.8% respectively). Interestingly, a high intra-individual variability in terms of anelloviruses abundance and composition over time was noticed; an increase in anelloviruses read counts as high as 10,000-fold was found between two consecutive time points (Figure 2b), and a turnover of the number and distribution of genogroups was observed during the course of the disease. In particular, an expansion of the TTV genogroup 4 abundance over time was noted for 7/10 patients (Figure 2b).

### 3.3. Correlation between mNGS and qPCR Assays for TTV

**Figure 3:**
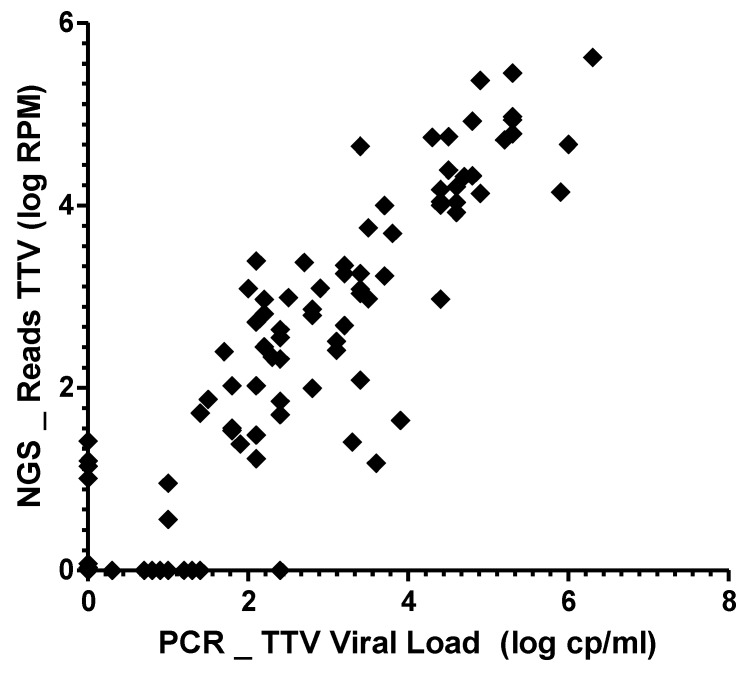
Correlation between the results of metagenomic NGS and TTV qPCR. Normalized number of reads obtained for torque teno virus (TTV) using mNGS are presented in function of the TTV viral load determined using qPCR. RPM: reads per million mapped reads.

To confirm mNGS results obtained for TTV, the 108 MM samples collected were also tested using a standardized TTV qPCR. TTV was detected in 81/108 (75.0%) samples and the median viral load was 2.3 log10 cp/mL. For HV, TTV was detected in 5/8 samples (median: 1.7 log10 cp/mL). The normalized number of TTV reads was strongly correlated with TTV load (Pearson correlation coefficient r = 0.91; *p* < 0.0001; Figure 3). As TTV represented the most abundant anellovirus detected in the plasma samples, the sex effect reported with mNGS testing was also noticed with qPCR (TTV viral load median for men = 3.9 log_10_ cp/mL vs. 1.0 log_10_ cp/mL for women, *p* < 0.0001).

## 4. Discussion

Using mNGS, we describe herein the individual anellovirus profile during the clinical course of 10 MM patients undergoing ASCT. Over recent years, the highly diverse anellovirus family has been identified as one of the main components of the human blood virome [2,14]. Among them, TTV, the first member discovered, is increasingly considered as a potential surrogate marker of immune competence [4,5]. Although TTV represented the most anellovirus detected in the present study, a high rate of coinfections involving TTMV and/or TTMDV was found, as previously reported, notably in hematological patients [9]. Interestingly, a study performed in pediatric lung transplant patients found that TTV levels were associated with short-term outcomes (acute rejection), while TTMV levels were associated with overall survival [15]. Taken together, this strongly suggests that anelloviruses other than TTV might also be of interest in transplantation medicine.

In addition, we confirmed a strong inter-individual variability regarding the abundance and diversity of anelloviruses [16]. In particular, the results emphasize that gender should be taken into account as a potential confounding factor to interpret anellovirus data. This marked sex effect, which is observed as soon as the time of diagnosis, has already been noticed [3,17] but not systemically included in studies investigating the use of anelloviruses as a biomarker [2]. This finding could be associated with the slower immune system decline reported in women than in men [18], however several other factors are also reported to interfere with the anellovirus cycle life, including age, levels of specific lymphocyte subsets, and cytomegalovirus immune status [3,5,17]. We also found a high level of turnover in anellovirus composition over time revealing a strong intra-individual variability. It has been shown that the number and distribution of TTV genogroups could be modified after the graft in both solid organ transplant patients and hematopoietic stem cell transplant recipients [19,20]. The results herein indicate that the change could occur within a short period of time, and thus, studies focusing on a specific time point should consider this information when interpreting results [21]. Many aspects of the anellovirus life cycle remain unclear but variable immune reconstitution among patients, inflammatory state underlying conditions could explain the difference in anellovirus profiles reported in the present study [8,19,22].

Altogether, the data presented herein does not support the hypothesis that a specific anellovirus is likely to be sufficient for the monitoring of patient immune competence as seems to be suggested by the large longitudinal study of solid organ transplant patients that found TTV-8 to be the main specie [2]; it is of note that this result was obtained after pooling samples from all the time points and did not take into account variability over time, but furthermore after pooling all samples herein it was found that TTV-16 was the most abundant species.

No significant correlation with clinical or laboratory parameters was found, which is in line with the limited number of patients enrolled. However, this small patient cohort allowed a comprehensive and individual characterization of the plasma virome dynamics. Furthermore, despite the homogeneity of patient management, we underscore the complex intra and inter-individual plasma virome variability in ASCT recipients. Although, more studies are needed to better understand the complex role of human virome in host health and disease, these findings are of prime importance for future investigations aiming to use anelloviruses as potential biomarkers in a clinical setting.

## Supplementary Materials

The following is available online at http://www.mdpi.com/1999-4915/10/11/633/s1, Figure S1: Individual kinetics of anellovirus reads proportion (among total viral reads) for the 10 ASCT recipients.
