# Supplementary material for: Metagenomic Next-Generation Sequencing Reveals Individual Composition and Dynamics of Anelloviruses during Autologous Stem Cell Transplant Recipient Management
Source: Viruses. 2018 Nov 14;10(11):633. doi: 10.3390/v10110633 (PMC6266913; doi:10.3390/v10110633)
Supplement: Supplementary file 1 [file viruses-10-00633-s001.pdf]

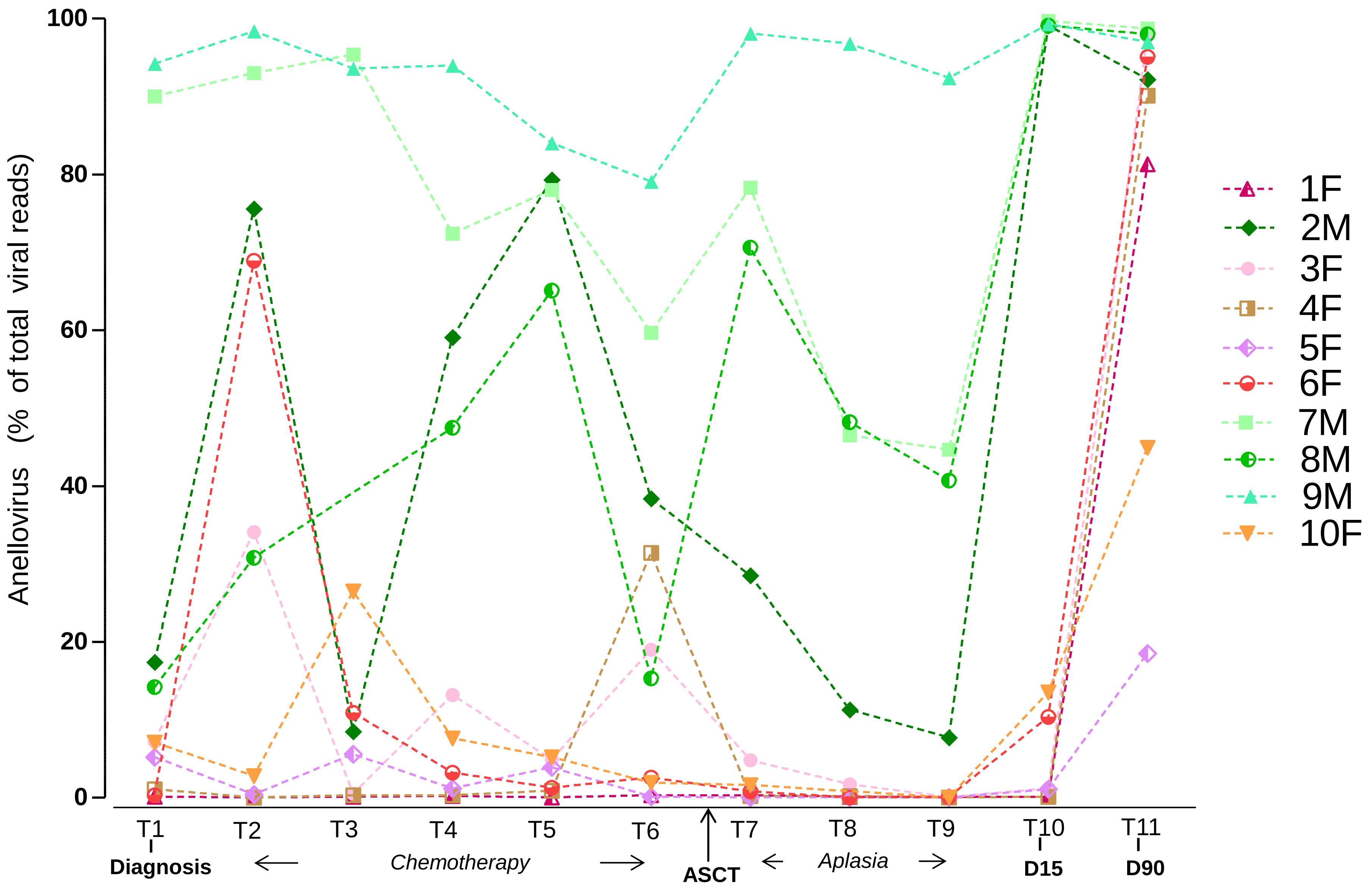

Figure S1. Individual kinetics of anellovirus reads proportion (among total viral reads) for the 10 ASCT recipients.
